# Identifying Novel ATX Inhibitors via Combinatory Virtual Screening Using Crystallography-Derived Pharmacophore Modelling, Docking Study, and QSAR Analysis

**DOI:** 10.3390/molecules25051107

**Published:** 2020-03-02

**Authors:** Ji-Xia Ren, Rui-Tao Zhang, Hui Zhang

**Affiliations:** 1 College of Life Science, Liaocheng University, Liaocheng 252059, China; 2 Institute of Medicinal Plant Development, Chinese Academy of Medical Science & Peking Union Medical College, 151 Malianwa North Road, Haidian District, Beijing 100193, China; 3 College of Agronomy, Liaocheng University, Liaocheng 252059, China; 4 College of Life Science, Northwest Normal University, Lanzhou 730070, China

**Keywords:** autotaxin inhibitor, 3D QSAR model, pharmacophore model, virtual screening, docking calculation

## Abstract

Autotaxin (ATX) is considered as an interesting drug target for the therapy of several diseases. The goal of the research was to detect new ATX inhibitors which have novel scaffolds by using virtual screening. First, based on two diverse receptor-ligand complexes, 14 pharmacophore models were developed, and the 14 models were verified through a big test database. Those pharmacophore models were utilized to accomplish virtual screening. Next, for the purpose of predicting the probable binding poses of compounds and then carrying out further virtual screening, docking-based virtual screening was performed. Moreover, an excellent 3D QSAR model was established, and 3D QSAR-based virtual screening was applied for predicting the activity values of compounds which got through the above two-round screenings. A correlation coefficient r^2^, which equals 0.988, was supplied by the 3D QSAR model for the training set, and the correlation coefficient r^2^ equaling 0.808 for the test set means that the developed 3D QSAR model is an excellent model. After the filtering was done by the combinatory virtual screening, which is based on the pharmacophore modelling, docking study, and 3D QSAR modelling, we chose nine potent inhibitors with novel scaffolds finally. Furthermore, two potent compounds have been particularly discussed.

## 1. Introduction

Autotaxin (ATX) is a circulating enzyme playing a primary role in the conversion of lysophosphatidyl choline (LPC) into the bioactive phospholipid derivative lysophosphatidic acid (LPA) [1,2].

Since the ATX-LPA signaling axis has been involved in a number of pathologies, including cancer [3,4,5,6,7], pain [8,9,10], and cholestatic pruritus [11,12], as well as fibrotic [13,14,15], inflammatory [16,17,18] and cardiovascular diseases [19], it attracts high interest in the drug discovery industry.

**Figure 1:**
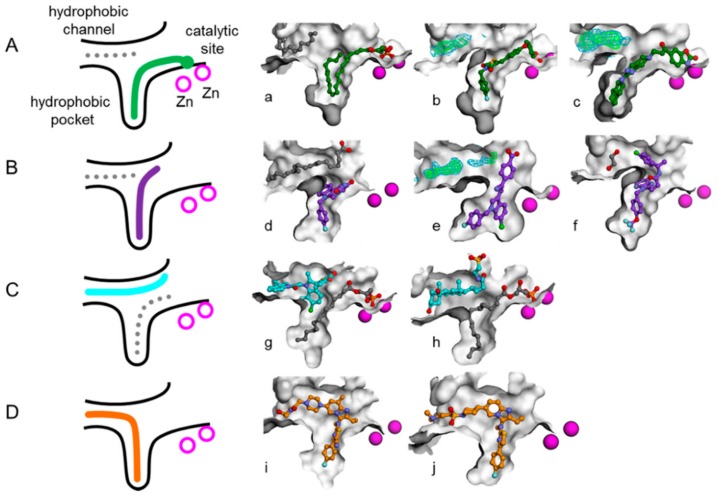
Schematic representation of the autotaxin (ATX) binding pocket and the different inhibitor binding modes reported to date. (This figure is cited from the reference 22) Type I, II, III, and IV inhibitors are represented in green, purple, cyan, and orange, respectively, on the schema with matching colors for carbon atoms on the exemplified structures. The gray dotted lines represent secondary ligands modeled next to the inhibitor as shown with gray carbons on the illustrations. Zinc ions are depicted in magenta. (**A**) Binding mode of type I inhibitors LPA (lysophosphatidic acid) 22:6 (a), HA-155 (b), and “compound 2”(c). (**B**) Binding mode of type II inhibitors PAT-494 (d), PAT-078 (e), and CRT0273750 (f). (**C**) Binding mode of type III inhibitors PAT-343 (g) and tauroursodeoxycholic acid (h). (**D**) Binding mode of type IV inhibitors GLPG1690 (i) and compound 9 (j).

**Figure 2:**
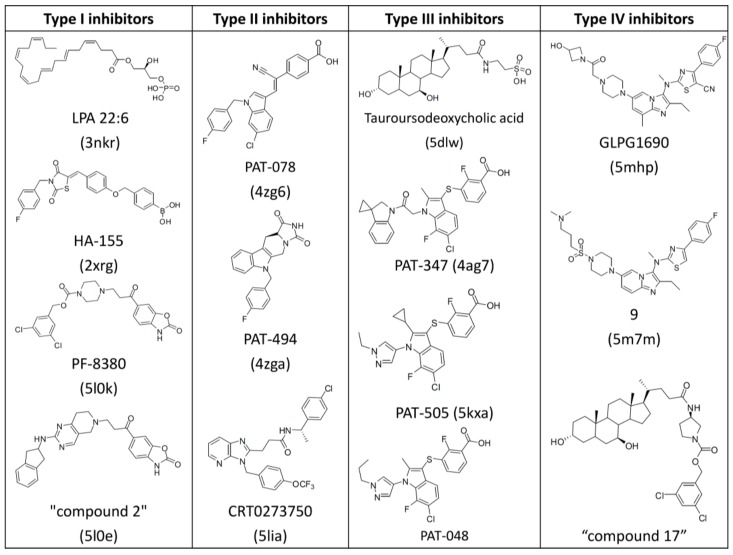
Chemical structures of representative ATX inhibitors. PDB codes of co-crystallized structures with ATX are indicated in brackets.

Recently, lots of patents and literature reported numerous ATX inhibitors with probable application for the treatment of diverse pathologies [10,14,20,21,22]. For example, Nicolas Desroy identified a first-in-class ATX inhibitor, GLPG1690, which has been undergoing clinical evaluation for the treatment of idiopathic pulmonary fibrosis [14]. An aminopyrimidine series with an ATX IC_50_ of 500 nM were developed by Spencer B. Jones, for the treatment of osteoarthritis pain [10]. The imidazo[1,2-a]pyridine series of ATX inhibitors were identified by the Nicolas Desroy and Bertrand Heckmann group [22]. According to the different binding modes of a variety of endogenous ATX ligands and synthetic ATX inhibitors to the active site of the ATX protein, the Nicolas Desroy and Bertrand Heckmann group classified the diverse structural inhibitors into four types, illustrated in Figure 1 [22]. Type I inhibitors mimic the binding mode of LPC substrate and occupy the catalytic site, as shown in Figure 1A. Type II inhibitors occupy the hydrophobic pocket by largely exploiting its intrinsic plasticity, as shown in Figure 1B. Type III inhibitors bind to the hydrophobic channel leaving the hydrophobic pocket and catalytic site unoccupied, as shown in Figure 1C. The binding modes of compounds studied in this research differ from that of other inhibitors and can therefore be categorized as type IV inhibitors, as shown in Figure 1D. Figure 2 manifests the represented compounds of four types of ATX inhibitors [22].

In this study, pharmacophore-based virtual screening (PB-VS) was first employed for retrieving new ATX inhibitors. We established pharmacophore models based on crystal structures of ATX-inhibitor complexes and then used a big test database to validate the developed pharmacophore models. Then, for discovering novel ATX inhibitors from the compounds passing through the PB-VS, docking-based virtual screening (DB-VS) was accomplished, and the score function and docking parameters were optimized before carrying out DB-VS. Finally, a good 3D QSAR model of 31 ATX inhibitors with excellent predictive ability was built and utilized to estimate the activity of compounds which have passed the above two-round screenings. QSAR-based virtual screening was performed to discover potential novel inhibitors with good inhibitory activities. After the above combinatory virtual screening method filtering, nine probable ATX inhibitors were chosen. We may buy them to accomplish the following activity experiments.

## 2. Materials and Methods

### 2.1. Development of Pharmacophore Models and PB-VS

The “Receptor-Ligand Pharmacophore Generation” protocol in Discovery Studio 3.1 (Accelrys Inc., San Diego, CA, USA) was applied to set up pharmacophore models. Some relevant parameters in this protocol were set as follows: assigning 6 to the “Maximum Feature”, assigning 4 to the “Minimum Feature”, and assigning 10 to the “Maximum Pharmacophore” [23]. In this study, two diverse crystal structures of the ligand-receptor complexes (PDB ID: 5MHP, 5M7M) were used for building the pharmacophore models of ATX inhibitors, because the two complexes manifest novel binding modes between inhibitors and the receptor [22]. Finally, we built 14 pharmacophore models. Through a big compound database, which includes 6396 decoy compounds (inactives) from the DrugBank database [24] and 34 ATX inhibitors (actives), the created pharmacophore models were discreetly verified.

We applied the well-built pharmacophore models as 3D queries to retrieve potential ATX inhibitors from the original chemical database “Diversity Libraries” (129,087 compounds, Life Chemicals Inc., Burlington, VT, Canada) by utilizing the “Search 3D Database” protocol in Discovery Studio 3.1.

### 2.2. Molecular Docking Calculation

We utilized GOLD 5.1 for performing the whole of the docking calculations in this investigation, which aimed at predicting affinities of compounds and interaction modes for the compounds getting through the PB-VS. The crystal structure (PDB ID: 5MHP) of ATX bound with the inhibitor 7NB was obtained for docking calculation. We put all hydrogen atoms into the ATX protein, and then used Discovery Studio 3.1 to distribute the CHARMM force field. The binding site was explored as a sphere which contains the amino acid residues staying within 12 Å from the ligand 7NB, and the binding site was big enough to overlay the ligand binding areas at the active site. Through docking these inhibitors, which are complexed with the ATX protein returning to their receptors’ active site, the score functions and docking parameters were pre-optimized.

### 2.3. Development of 3D QSAR Model and QSARB-VS

A total of 34 ATX inhibitors were collected [22] and docked into the ATX’s active site to explore the possible binding conformations, and then the inhibitors with probable binding poses were superimposed with the “Molecular Overlay” tool. However, three compounds with obviously wrong binding conformations were deleted from the 34 inhibitors, therefore, a total of 31 aligned inhibitors were used for establishing a 3D QSAR model. Seventy percent (22 compounds) of them, as shown in Table S1, were used as a training set to build the 3D QSAR model, and then the remnant 30% (including 9 compounds), as shown in Table S2, were used as an outer test set for verifying the predictive ability of the 3D QSAR model.

The “Generate Training and Test Data” protocol in Discovery Studio 3.1 was utilized to generate the training set compounds and test set compounds by using the “random” method.

At first, we made the compound’s inhibitory activity in reports [IC_50_ (nmol/L)] to become the negative log scale [pIC_50_ (mol/L)], which was employed as the responding variable for the following 3D QSAR analysis.

The CHARMM force field was added. The van der Waals potential, combined with the electrostatic potential, were treated as individual terms in Discovery Studio 3.1. A + le point change was applied as the electrostatic potential probe when the dielectric constant related to distance was for mimicking the effect of solvent. Concerning the van der Waals potential, a carbon atom was used, whose radius equaled 1.73 Å, as the probe [25]. We utilized energy grids as signifiers to build a partial least-squares model and used the “Create 3D QSAR Model” protocol in Discovery Studio 3.1 to establish 3D QSAR models.

We carried out the QSARB-VS by utilizing the “Calculate Molecular Properties” protocol. The selected final hits were the compounds whose estimated pIC_50_ value were higher than 5.6.

## 3. Results and Discussion

### 3.1. Establishment of Pharmacophore Models

**Figure 3:**
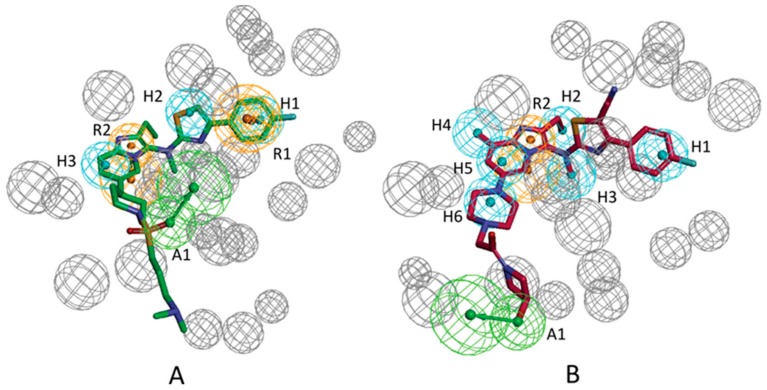
(**A**) All chemical features identified based on the 5M7M complex. (**B**) All chemical features identified based on the 5MHP complex. Feature colors: blue, hydrophobic feature; green, hydrogen acceptor feature; orange, aromatic ring feature.

By utilizing the “Receptor-Ligand Pharmacophore Generation” protocol, the pharmacophore models from the crystal ATX-ligands were derived. The pharmacophore generation module in Discovery Studio 3.1 interprets and abstracts chemical properties, which include charge properties, hydrogen acceptor, hydrophobic feature, hydrogen donor, and aromatic feature from the receptor–ligand interactions. Several excluded volume spheres and chemical properties were produced and perceived as pharmacophore models; these models can be applied for discovering small molecular compounds with the capacity of inhibiting ATX activity. For ATX-7HR (PDB ID: 5M7M), the software recognized four pharmacophore models, which were termed as 5M7M 01–04, and for ATX-7NB (PDB ID: 5MHP), 10 pharmacophore models, which were referred to as 5MHP 01–10, were recognized by the software. Figure 3A manifests the excluded volume spheres and the whole pharmacophore properties derived from the mutual interactions between ligand 7HR and the ATX receptor. Figure 3B manifests the whole pharmacophore properties and excluded volume spheres derived from the mutual effects between ligand 7NB and the ATX receptor. Table 1 manifests the pharmacophore summary of the pharmacophore models 5M7M 01–04 and 5MHP 01–10. The selectivity scores are employed for ranking the pharmacophore models. The detailed information about calculation of the selectivity score can refer to reference [23]. The selectivity score is estimated based on a genetic function approximation (GFA) model. The GFA model for the selectivity of a pharmacophore is built from a training set of 3285 pharmacophore models. This set is used for searching the CapDiverse database in Discovery Studio. The logarithmic values of the number of database search hits are used as the targets (a value of −1.0 is used if no hit is retrieved from the search). The number of total features in pharmacophore models and the feature–feature distance bin values are used as the descriptors for training the GFA model.

### 3.2. Validation of Pharmacophore Models

By utilizing a big test database (including 6396 compounds which were from the DrugBank database [24] and 34 ATX inhibitors [22]), we carefully validated the total of 14 pharmacophore models for their ability of determining external compounds as ATX inhibitors or ATX non-inhibitors. The parameters which were employed to evaluate the prophetic capability of pharmacophore models are as follows: specificity, (SP (1)), the predictive accuracy of the ATX non-inhibitors); sensitivity (SE (2)), the predictive accuracy of the ATX inhibitors); the total predictive accuracy (Q (3)).

(1) $$\mathrm{SP}=\frac{\mathrm{TN}}{\mathrm{TN}+\mathrm{FP}}$$

(2) $$\mathrm{SE}=\frac{\mathrm{TP}}{\mathrm{TP}+\mathrm{FN}}$$

(3) $$Q=\frac{\mathrm{TP}+\mathrm{TN}}{\mathrm{TP}+\mathrm{TN}+\mathrm{FP}+\mathrm{FN}}$$

FP, which means false positives, is the number of ATX non-inhibitors which are incorrectly predicted as ATX inhibitors; TN, which means true negatives, is the number of properly identified ATX non-inhibitors; TP, which means true positives, is the number of properly confirmed ATX inhibitors; FN, which means false negatives, is the number of ATX inhibitors that are incorrectly categorized as ATX non-inhibitors.

The ROC score is described as the area under the ROC curve (AUC), which is widely used to measure the discriminatory power of a pharmacophore model. For example, the maximum value for ROC, which equals 1, manifests that the model has an ideal predictive capability, which means a 0% wrong positive rate and a 100% real positive rate. However, if the ROC score is lower than 0.5, it manifests that the model has no discriminative capacity, which means a 50% wrong positive rate and a 50% real positive rate [26].

Table 2 displays the prediction results of the test set for the total 14 pharmacophore models. As can be seen from Table 2, for the four pharmacophore models which were established based on 5M7M complex, the ROC scores range from 0.770 to 0.845. (ROC curves of models 5M7M 01–04 are displayed in Figure S1). The values of sensitivity (SE), as shown in Table 2, are all 1; however, the values of specificity (SP), as shown in Table 2, range from 0.624 to 0.741. Only one pharmacophore model, 5M7M 01, has the value of SP of 0.741 and the concordance (Q), as shown in Table 2, of 0.742; the numerical values of Q and SP of the rest of the pharmacophore models are all lower than 0.700. Therefore, we only selected the pharmacophore model 5M7M 01 for the virtual screening. For the ten pharmacophore models established based on the 5MHP complex, except for 5MHP 01, 02, 04, and 05, the ROC scores are all higher than 0.900 (ROC curves of models 5MHP 01–10 are displayed in Figure S2), which manifests the ability of the models to distinguish ATX inhibitors from non-inhibitors is excellent. The values of SE of pharmacophore models 5MHP 01, 02, and 05 are all lower than 0.800; these results were not entirely satisfactory, so these three models were not applied for the virtual screening. The rest of the pharmacophore models possess good SE, SP, and ROC values; therefore these pharmacophore models would be employed for virtual screening. The ten pharmacophore models have the concordance (Q), as shown in Table 2, ranging from 0.881 to 0.971.

### 3.3. Determination of Parameters and Scoring Functions

**Figure 4:**
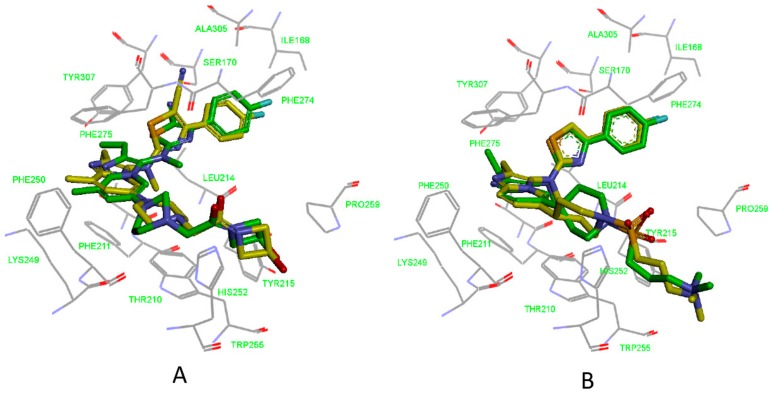
Binding modes of 7HR (yellow) (**A**) and 7NB (yellow) (**B**) in the active site of ATX. Ligands complexed with their receptors are also shown for comparison, 7HR and 7NB indicated in green stick form.

As mentioned above, prior to carrying out the virtual screening, the QSARB-VS and PB-VS needed to establish virtual screening models. Compared to QSARB-VS and PB-VS, if the receptor crystal structure is known, DB-VS seems straightforward. Because the score functions and docking parameters have been deemed to have significant effects on the ultimate results of DB-VS, including affinities between compounds and receptor and binding conformations of compounds, it is necessary to optimize the score functions and docking parameters before carrying out the real DB-VS. In the research, we utilized GOLD 5.1 for the DB-VS, which has been deemed as one of the greatest programs of docking software. The reference structure of the receptor for the docking calculation was the crystal structure (PDB ID: 5MHP) of the ATX-7NB complexes. Two active compounds co-crystallizing with ATX were docked to return to the ATX’s active site for determining the optimal docking parameters and score functions. The docking parameters and score functions were adjusted before the docked conformations approach their initial crystallized conformations as much as possible. The ultimately optimized docking parameters were maintained as their set default, besides that, the genetic algorithm parameter was assigned to “GOLD Default”; the early termination was assigned to “False”; and generate diverse solutions was assigned to “True”. To rank the compounds, the Chemscore fitness function was chosen. By using these parameters and score function, we acquired very little root-mean-square deviation (RMSD) values between the docked conformations of the two active compounds and their crystal conformations. Figure 4 shows the docking poses of the 7HR and 7NB, for comparison, and the crystal structures of the two compounds that have been complexed with ATX are also shown. An overview is that the docked structures (both the poses and positions of heavy atoms) are very close to their original crystallized structures. The computed RMSD values are displayed in Table 3. Clearly, the compound 7HR has the RMSD value of 1.8001 Å, and the other compound 7NB owns the RMSD value of 0.6007 Å. The two compounds possess RMSD values less than 2.0 Å, showing that GOLD software is a reliable way for docking computations and capable of searching the right conformations.

### 3.4. Development of the 3D QSAR Model

**Figure 5:**
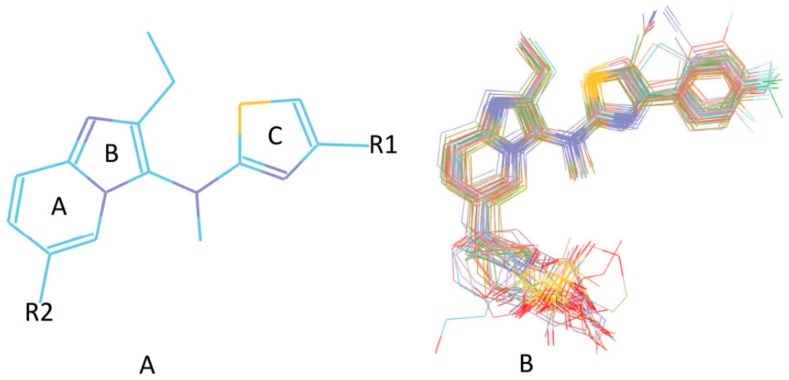
(**A**) The scaffold of 31 autotaxin inhibitors. (**B**) The alignment result of 31 Mcl-1 inhibitors based on the poses acquired by the docking study calculation.

**Figure 6:**
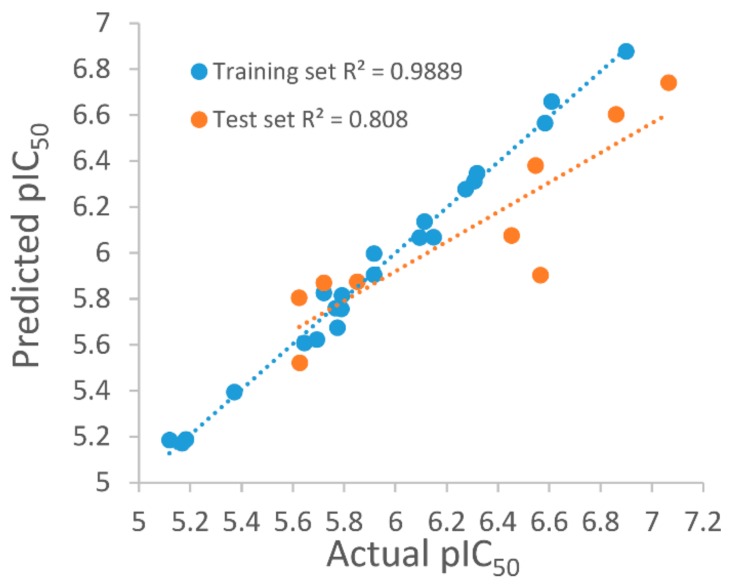
Plots of predicted autotaxin inhibitory activities versus experimental of training set and test set.

For the purpose of obtaining a structure–activity relationship profile on the compound N-N,4-dimethylthiazol-2-amine derivates as ATX inhibitors and of retrieving potential ATX inhibitors through the VS way, we built 3D QSAR models. The greatest 3D QSAR model was utilized to evaluate the pIC_50_ values of new compounds. In the above work, 31 ATX inhibitors bearing the same scaffold, as shown in Figure 5A, with experimental IC_50_ values were gathered as the 3D QSAR dataset. The “Generate Training and Test Data” protocol in Discovery Studio 3.1 was applied to generate the training and test sets by using the “random” method. Take into consideration, a good alignment of compounds used for QSAR modeling is important for molecular field analysis, and the whole 34 compounds were docked into the ATX’s active site to probe each compound’s probable binding conformation, and three compounds were deleted after checking their binding conformations since these compounds possessed obviously wrong binding conformations. Then, the remaining 31 compounds which have binding conformations were superimposed by utilizing the “Molecular Overlay” tool, and the 3D QSAR model was developed by making use of the 31 aligned inhibitors. Figure 5B presents the alignment result of the 31 ATX inhibitors. The correlation coefficient r^2^ between estimated and experimental activity of the training set was identified as 0.988, while the correlation coefficient r^2^ of the test set was identified as 0.808, manifesting that the established 3D QSAR model was an excellent model for exploring the QSAR of the 31 inhibitors. Figure 6 shows the good agreement between predicted pIC_50_ values and experimental pIC_50_ values for both the test set and training set.

**Figure 7:**
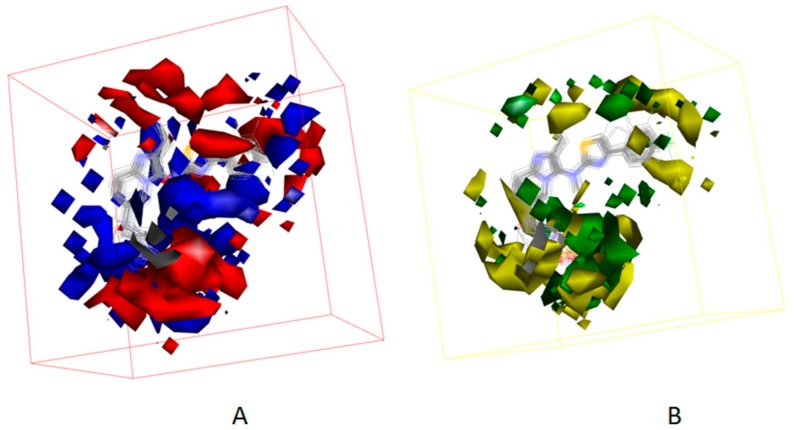
(**A**) 3D QSAR model coefficients on electrostatic potential grids. Blue represents positive coefficients; red represents negative coefficients. (**B**) 3D QSAR model coefficients on van der Waals grids. Green represents positive coefficients; yellow represents negative coefficients.

Besides, Figure 7 displayed the compounds which correspond with the iso-surface of the 3D QSAR model coefficients on the electrostatic potential grids and van der Waals grids. In the electrostatic map, red contours surround the areas where high electron density is expected to enhance activity, and blue contours describe areas where low electron density is expected to enhance activity. Similarly, the steric map indicates areas where steric bulk is predicted to increase (green) or decrease (yellow) activity. On the basis of the mappings, there are no locations of electrostatic potential grids and van der Waals grids for both ring A and ring B, manifesting that those locations are not crucial for increasing the activities of compounds, but proper core minor structures are required. However, the meta-position of ring B needs small substitutional and negative charged groups, and there are no distributions of van der Waals grids, but a negative charged group distribution for ring C. The substitutional groups, which are in the R1 site, demand high negatively charged groups on the aromatic ring, and the small substitutional groups on the rings. The substitutional groups in the R2 site demand small substitutional groups and high negatively charged groups. Consequently, data which was summed up proves that compound**23**, which has a proper substituted group and is the greatest potent ATX inhibitor, has outstanding activity, with an experimental IC_50_ value equal to 86 nM.

### 3.5. Searching for New ATX Inhibitors

**Figure 8:**
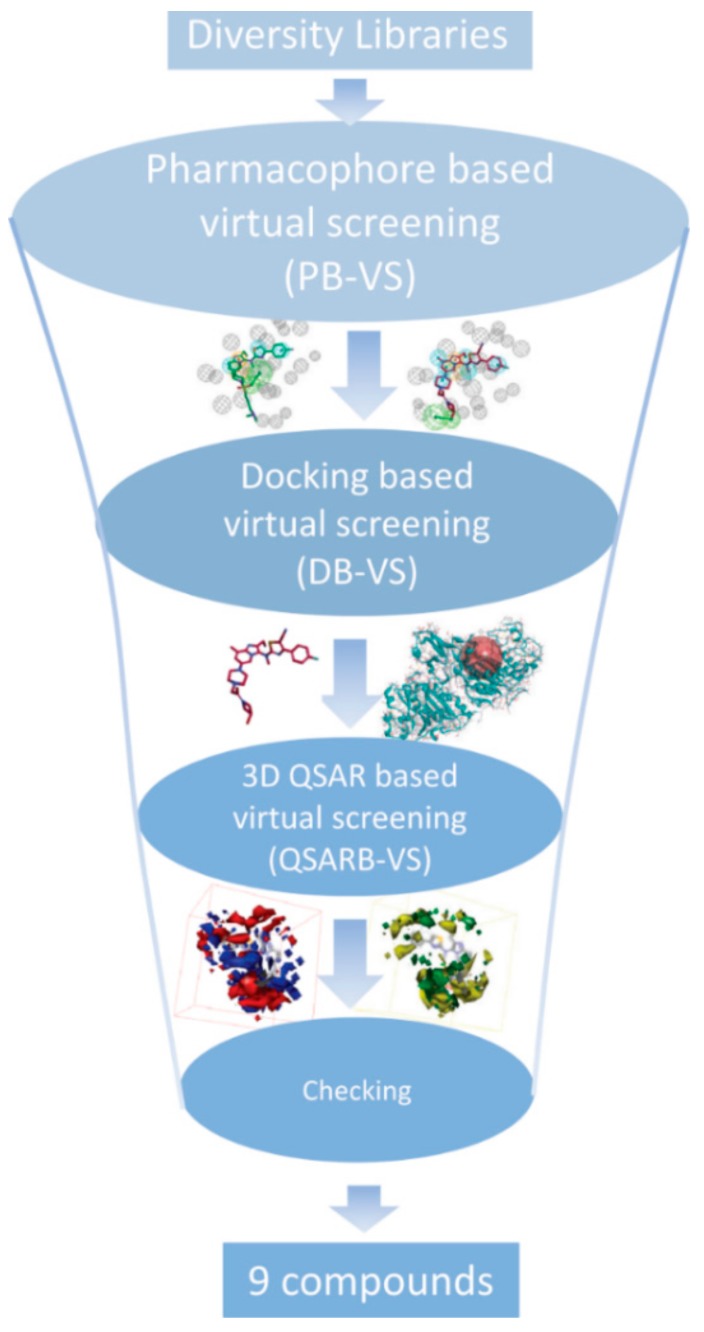
The workflow chart of the study. A combinatory virtual screening (VS) protocol based on the pharmacophore model, molecular docking study, and the 3D QSAR model was utilized to discover novel inhibitors targeting autotaxin.

**Figure 9:**
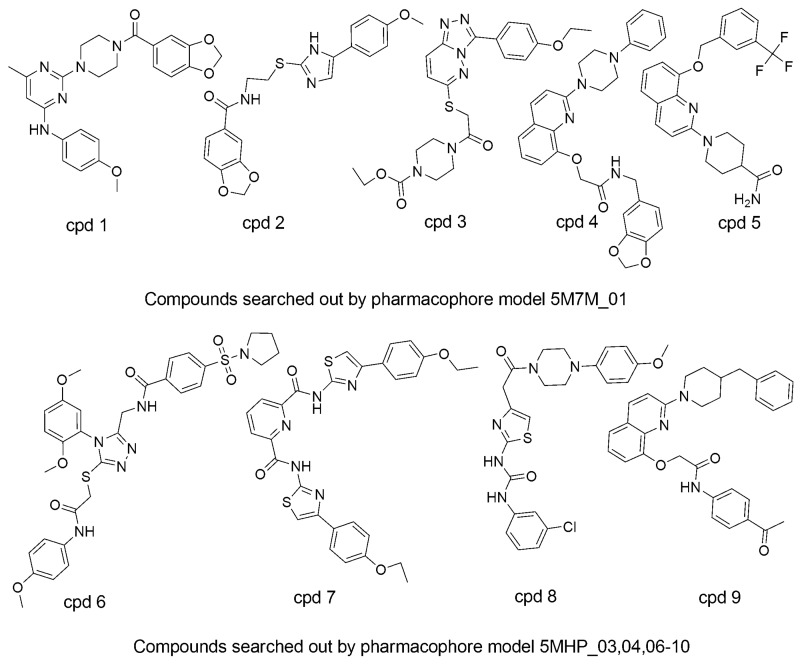
The 3D chemical structures of the final nine selected compounds.

In order to find patent ATX inhibitors, we conducted a combined virtual screening method, including a 3D QSAR model, molecular docking calculations, and pharmacophore models [27]. Figure 8 manifests the virtual screening workflow. Using the PB-VS filter, the original chemical database was first filtered, for PB-VS was quicker than DB-VS. Nevertheless, the pharmacophore models which were utilized in the research only thought of the chemical properties between the receptor and the ligand, and these models did not have the capacity to estimate the inhibitory activities of the potent compounds. Consequently, the hit compounds retrieved by PB-VS were docked to the active site of ATX receptor for sorting these compounds and discovering rational binding conformations of those compounds to do some preparation for the activity prediction by the 3D QSAR model. It should be emphasized that the 14 pharmacophore models applied in the research are non-quantitative structure–activity relationship pharmacophore models, and those models do not involve the activity-relationship of the ATX inhibitors and cannot evaluate activity of patent compounds. Thus, the 3D QSAR model, which can predict the inhibitory activity of new compounds, was finally applied to filter the selected compounds passing through the two rounds of selection, which includes DB-VS and PB-VS. In detail, when the cutoff fitting value is assigned as 2.0, 2846 compounds could get over the PB-VS, and the 2846 compounds could be sorted by utilizing the docking computation. On the basis of the docking scores and whether there are some significant mutual effects existing between the selected compounds and the ATX protein’s active position (including PHE211, LEU214, PHE274, PHE275, ALA305274, and TYR307), 50 compounds were selected. Then, the 3D QSAR model was used to filter 50 compounds which have probable binding conformations, and nine compounds were selected whose estimated pIC_50_ values were higher than 5.6. Figure 9 manifests the chemical structures of the nine chosen compounds. Table 4 stands for the nine hit compounds’ relevant parameters, which includes pharmacophore fit values, docking scores, and pIC_50_ values.

**Figure 10:**
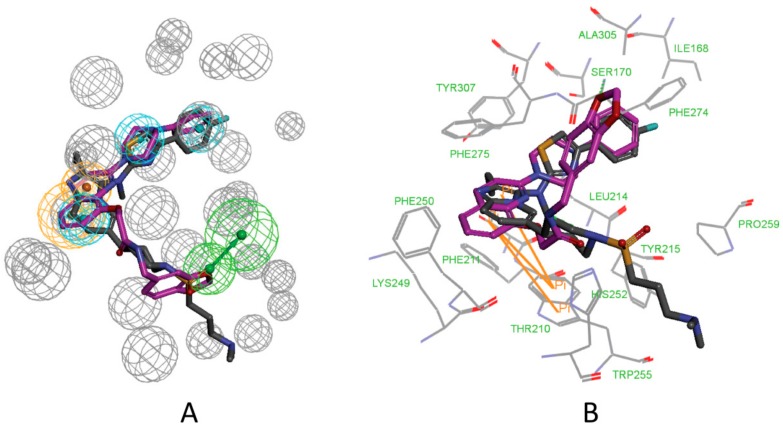
(**A**) Mapping of 5M7M 01 with compound cpd4. (**B**) The possible binding pose of cpd1 in the autotaxin active site. Compound 7HR complexed with autotaxin is also shown for comparison (in gray stick form).

From Figure 10A, we can conclude that the features of the pharmacophore model 5M7M 01 are mapped well with one hit compound cpd4. In detail, the phenyl groups and piperazine group of cpd4 map with the three hydrophobic features. The oxygen atom of the benzo[d][1,3]dioxole group maps with the hydrogen acceptor feature. The phenyl group maps with the ring aromatic feature. Figure 10B shows the probable binding conformation of cpd4 in the ATX’s active site; the 1-phenylpiperazine group forms hydrophobic interactions with the residues ILE168, LEU214, PHE274, PHE275, and ALA305; the oxygen atom of benzo[d][1,3]dioxole group forms hydrogen bond interactions with the SER170; the quinoline group makes π-π interactions with TRP255. 

**Figure 11:**
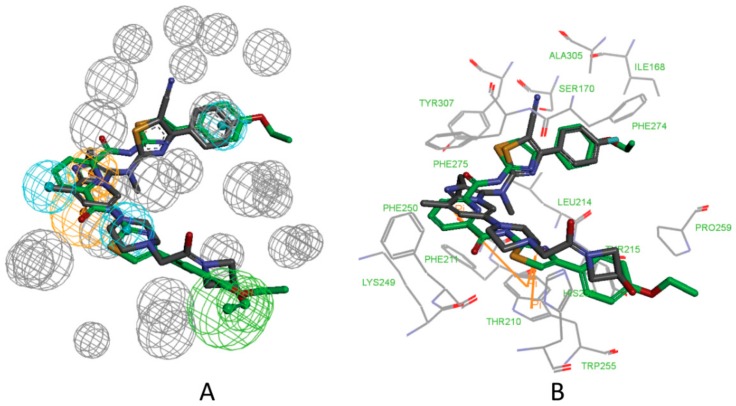
(**A**) Mapping of 5MHP 03 with compound cpd7. (**B**) The possible binding pose of cpd7 in the autotaxin active site. Compound 7NB complexed with autotaxin is also shown for comparison (in gray stick form).

The mapping result of the compound cpd7 with the pharmacophore model 5MHP 03 is shown in Figure 11A. The hydrophobic features map with the phenyl group and thiazole group; the oxygen atom of methoxyethane group maps with the hydrogen acceptor feature, and unfortunately, in Figure 11B, the corresponding hydrogen bonds disappear. The pyridine group maps with the aromatic ring feature. Figure 11B shows the probable binding conformation of the compound cpd7 in the active site of ATX. The 4-(4-ethoxyphenyl)thiazole group of cpd7 forms hydrophobic interactions with the amino acid residues ILE168, LEU214, PHE274, PHE 275, and ALA305. The pyridine group and thiazole group make π-π interactions with TRP255. 

**Figure 12:**
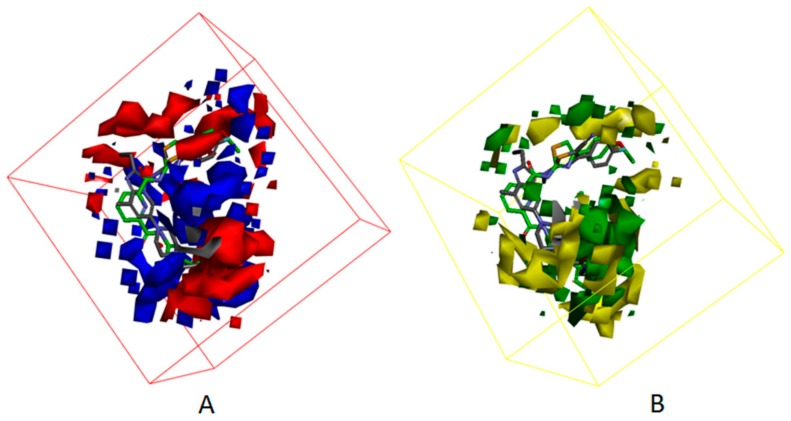
The mappings of cpd7 with isosurface-EP (–, red; +, blue) (**A**), isosurface-VMD (–, yellow; +, green) (**B**) grids. cpd7 is presented in green stick form. Compound 7NB complexed with autotaxin is also shown for comparison (in gray stick form).

**Figure 13:**
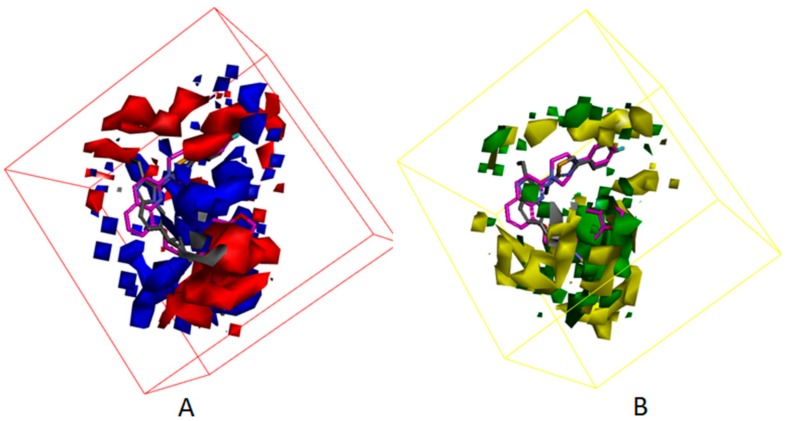
The mappings of cpd4 with isosurface-EP (–, red; +, blue) (**A**), isosurface-VMD (–, yellow; +, green) (**B**) grids. cpd4 is presented in purple stick form. Compound 7HR complexed with autotaxin is also shown for comparison (in gray stick form).

Figure 12 and Figure 13 show the mapping results of cpd4 and cpd7 with the 3D QSAR model, respectively. The mapping results of cpd4 and cpd7 with electrostatic potential grids are shown in Figure 12A and Figure 13A; Figure 12B and Figure 13B show the mapping results of cpd4 and cpd7 with van der Waals grids. From the figures, we can conclude that the cpd4 and cpd7 map well with the 3D QSAR model; if the 1-phenylpiperazine group of cpd4 and ethoxybenzene group of cpd7 are substituted by heterocyclic or aromatic rings with negative charge and a small substituent group, the activities of cpd4 and cpd7 may be increased.

## 4. Conclusions

In the research, we first established 14 pharmacophore models of N-N,4-dimethylthiazol-2-amine derivates as ATX inhibitors by utilizing the “Receptor–Ligand Pharmacophore Generation” protocol. The 14 pharmacophore models were derived from the 5MHP and 5M7M ligand–receptor complexes. By utilizing a big database which includes 6396 decoys and 34 ATX inhibitors, the pharmacophore models were then validated. Next, we used a docking study to execute virtual screening. We obtained the appropriate score function and docking parameters before performing the actual virtual screening through estimating the values of RMSD between the ligands’ crystal postures and the docked conformations of them. By utilizing the 31 aligned ATX inhibitors, we built a remarkable 3D QSAR model, and the corresponding coefficient r^2^ between estimated and experimental activities (the estimated activities were predicted by the 3D QSAR model) of the test set and training set compounds were 0.808 and 0.988, separately. Next, a combined virtual screening method was utilized to filter patent ATX inhibitors, which included pharmacophore models, molecular docking calculation, and 3D QSAR model approaches. At first, we used the pharmacophore models to screen the initial database. Secondly, we docked the hit compounds into the active site of ATX to predict their probable binding postures. Lastly, to find feasible patent ATX inhibitors, we employed the 3D QSAR model to predict the activities of the compounds through the two-round filterings. We discreetly selected nine feasible ATX inhibitors and may buy them to proceed to the following tests.

## Figures and Tables

**Table 1:** The summary of pharmacophore models 5M7M 01–04.

Pharmacophore Abstract			
Pharmacophore Models	Frequency of Properties	Feature Set	Selectivity Score
5M7M 01	5	A1H1H2H3R2	1.3840
5M7M 02	4	A1H1H2R2	1.0423
5M7M 03	4	A1H3R1R2	0.95229
5M7M 04	4	A1H1H2H3	0.47716
5MHP 01	6	A1H1H2H3H4H5	3.6354
5MHP 02	6	A1H1H2H3H4H6	3.6354
5MHP 03	6	A1H1H2H4H6R2	2.8903
5MHP 04	6	A1H1H3H4H6R2	2.8903
5MHP 05	5	A1H1H3H4H5	2.4170
5MHP 06	5	A1H1H2H3H4	2.4170
5MHP 07	5	A1H1H2H4H5	2.4170
5MHP 08	5	A1H1H3H4H6	2.4170
5MHP 09	5	A1H1H2H4H6	2.4170
5MHP 10	5	A1H1H4H6R2	2.3270

**Table 2:** The verification results of pharmacophore models 5MHP 01–10 and 5M7M 01–04.

Verification with Known Inactives/actives										
Pharmacophore	TA*^a^*	TI*^b^*	TP*^c^*	TN*^d^*	FP*^e^*	FN*^f^*	SE*^g^*	SP*^h^*	Q*^i^*	ROC
5M7M 01	34	6396	34	4737	1659	0	1	0.741	0.742	0.845
5M7M 02	34	6396	34	4108	2288	0	1	0.642	0.6442	0.770
5M7M 03	34	6396	34	4002	2394	0	1	0.626	0.628	0.793
5M7M 04	34	6396	34	3991	2405	0	1	0.624	0.626	0.773
5MHP 01	34	6396	19	6172	224	15	0.559	0.965	0.963	0.766
5MHP 02	34	6396	26	6219	177	8	0.765	0.972	0.971	0.866
5MHP 03	34	6396	33	6093	303	1	0.971	0.953	0.953	0.967
5MHP 04	34	6396	28	6155	241	6	0.824	0.962	0.962	0.894
5MHP 05	34	6396	25	5773	623	9	0.735	0.903	0.902	0.829
5MHP 06	34	6396	34	5634	762	0	1	0.881	0.881	0.969
5MHP 07	34	6396	34	5590	806	0	1	0.874	0.875	0.943
5MHP 08	34	6396	32	5845	551	2	0.941	0.914	0.914	0.927
5MHP 09	34	6396	34	5788	608	0	1	0.905	0.905	0.959
5MHP 10	34	6396	34	5650	746	0	1	0.883	0.884	0.951

*^a^* True actives; *^b^* true inactives; *^c^* true positives; *^d^* true negatives; *^e^* false positives; *^f^* false negatives; *^g^* sensitivity; *^h^* specificity; *^i^* concordance.

**Table 3:** The crystal ATX inhibitors’ RMSD values between their docked postures and crystal postures.

Ligand ID*^a^*	PDB Entry	RMSD (Å)
7HR	5M7M	1.8001
7NB	5MHP	0.6007

*^a^* The ligand IDs are from the PDB database.

**Table 4:** The nine hit compounds’ parameters, which include docking score, pharmacophore fit values, and pIC_50_ values.

Compound	Fit Value	Docking Score	Predicted Activity (pIC_50_)
cpd1	2.95497	37.0191	5.6312
cpd2	2.33815	43.5524	5.63735
cpd3	2.08654	34.011	5.687
cpd4	2.00695	48.8647	5.62577
cpd5	3.63318	42.5693	5.71689
cpd6	3.55526	34.2583	5.75721
cpd7	3.14614	33.264	5.74321
cpd8	3.12769	40.4997	5.68862
cpd9	2.53449	35.5562	5.76814

## Supplementary Materials

The following are available online.
