# Supplementary material for: Identifying Novel ATX Inhibitors via Combinatory Virtual Screening Using Crystallography-Derived Pharmacophore Modelling, Docking Study, and QSAR Analysis
Source: Molecules. 2020 Mar 2;25(5):1107. doi: 10.3390/molecules25051107 (PMC7179221; doi:10.3390/molecules25051107)
Supplement: Supplementary file 1 [file molecules-25-01107-s001.pdf]

molecules

Identifying Novel ATX Inhibitors via Combinatory Virtual Screening Using Crystallography-Derived Pharmacophore Modelling, Docking Study, and QSAR Analysis

Ji-Xia Ren [1,2,*,†], Rui-Tao Zhang [3,†] and Hui Zhang [4]

1 College of Life Science, Liaocheng University, Liaocheng 252059, China
2 Institute of Medicinal Plant Development, Chinese Academy of Medical Science & Peking Union Medical college, 151 Malianwa North Road, Haidian District, Beijing 100193, China
3 College of Agronomy, Liaocheng University, Liaocheng 252059, China; zhangrt86@foxmail.com
4 College of Life Science, Northwest Normal University, Lanzhou 730070, China; zhanghui123gansu@163.com
* Correspondence: renjixia@lcu.edu.cn
† These authors contributed equally.

Supplementary Materials

Table S1. Chemical structures of the 22 training set inhibitors for building the 3D QSAR model with their IC_{50} and pIC_{50} values.

Compound code	Chemical structure	IC_{50} (nM)	pIC_{50}
1	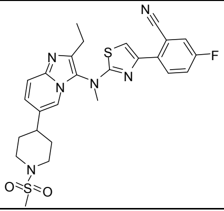	126	6.89963
2	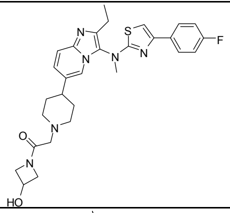	246	6.60907
3	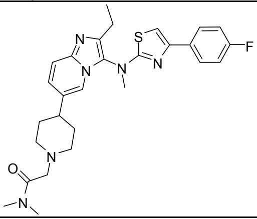	261	6.58336
4	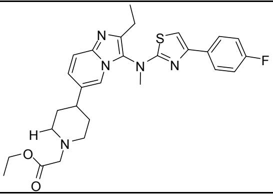	480	6.31876
5	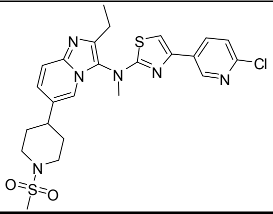	493	6.30715

6	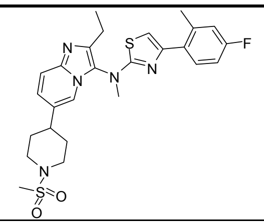	533	6.27327
7	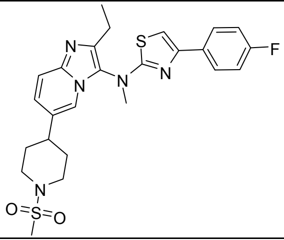	710	6.14874
8	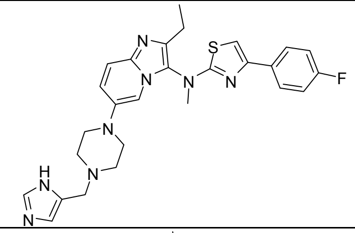	771	6.11295
9	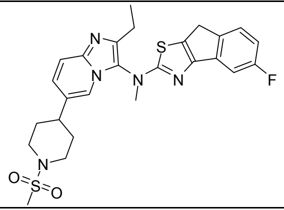	804	6.09474
10	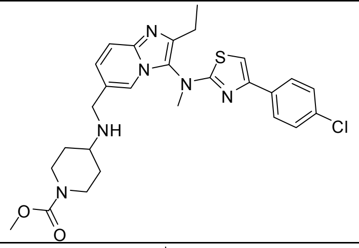	1,210	5.91721
11	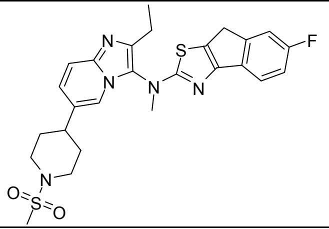	1,212	5.9165
12	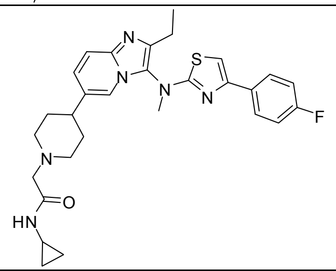	1,614	5.7921
13	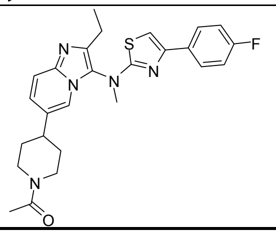	1,624	5.78941

14	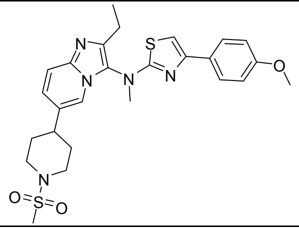	1,684	5.77366
15	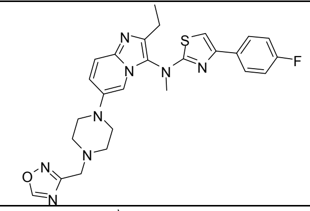	1,708	5.76751
16	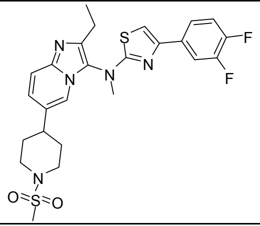	1,899	5.72148
17	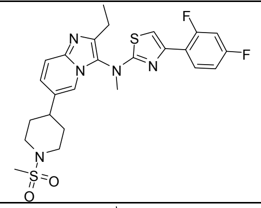	2,021	5.69443
18	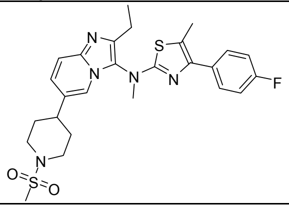	2,266	5.64474
19	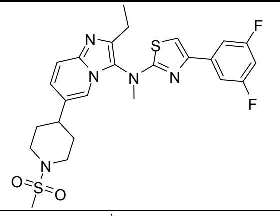	4,246	5.37202
20	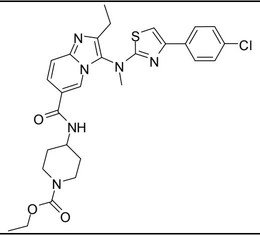	6,555	5.18343
21	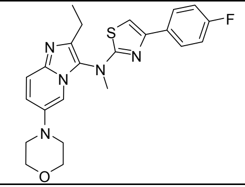	6,800	5.16749
22	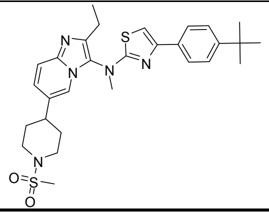	7,609	5.11867

Table S2. Chemical structures of the 9 test set inhibitors for building the 3D QSAR model with their IC_{50} and pIC_{50} values.

Compound code	Chemical structure	IC_{50} (nM)	pIC_{50}
23	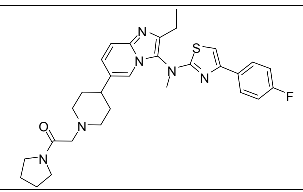	86	7.0655
24	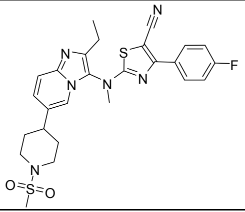	138	6.86012
25	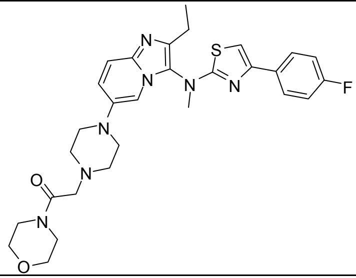	231	6.63639
26	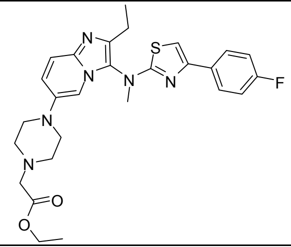	280	6.55284
27	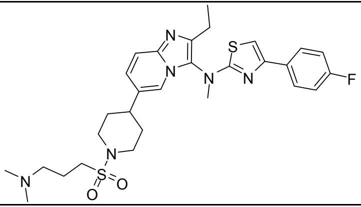	357	6.44733
28	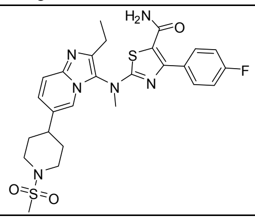	1,410	5.85078
29	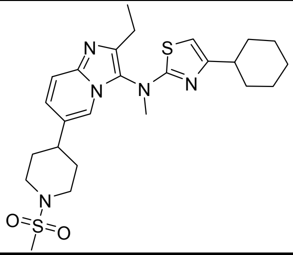	1,904	5.72033

30	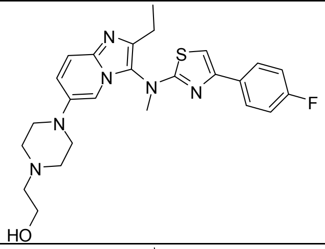	2,359	5.62727
31	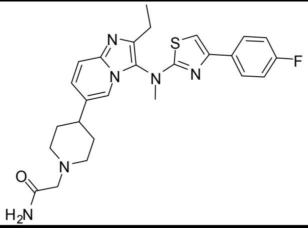	2,372	5.62489

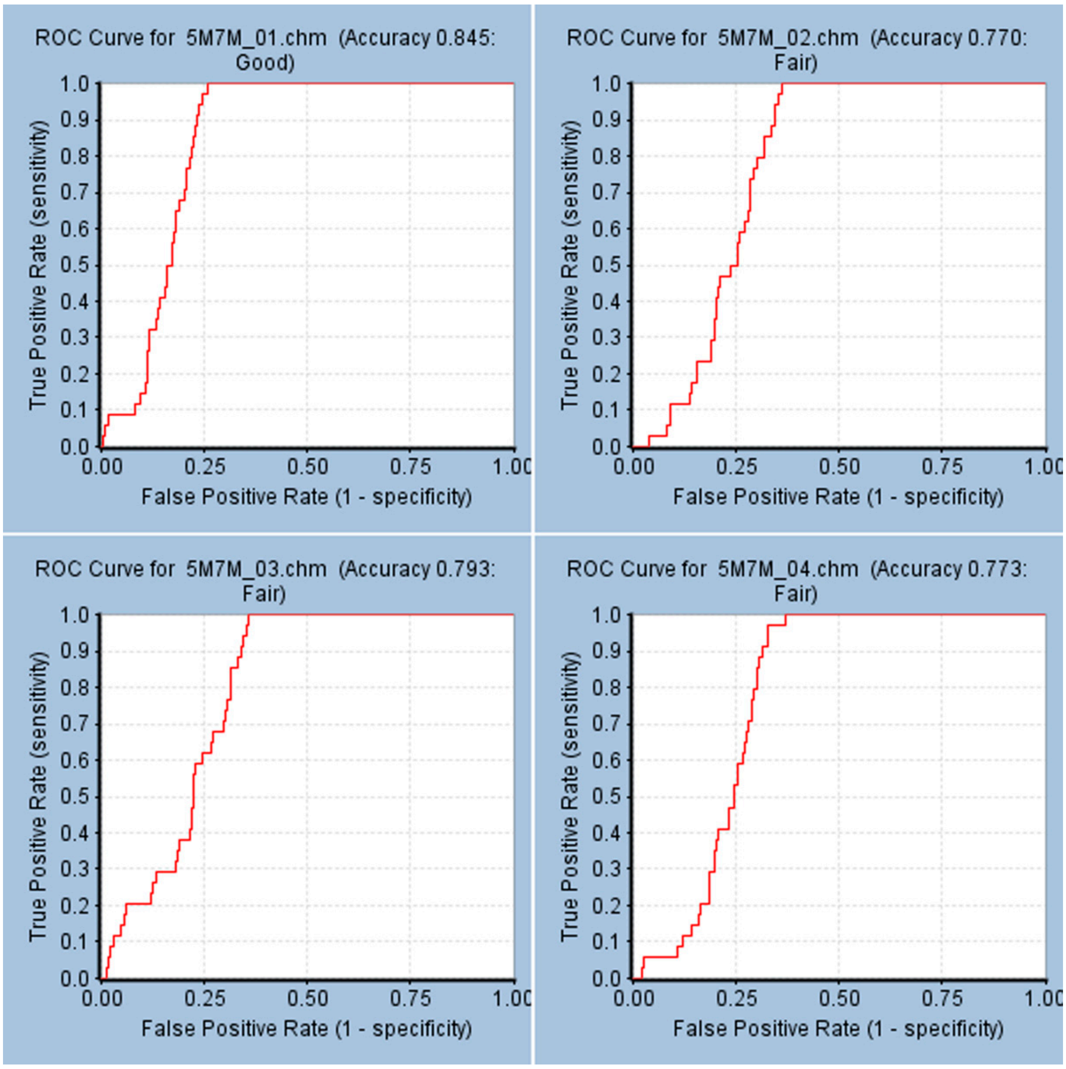

Figure S1. ROC curves of pharmacophore models 5M7M 01-04.

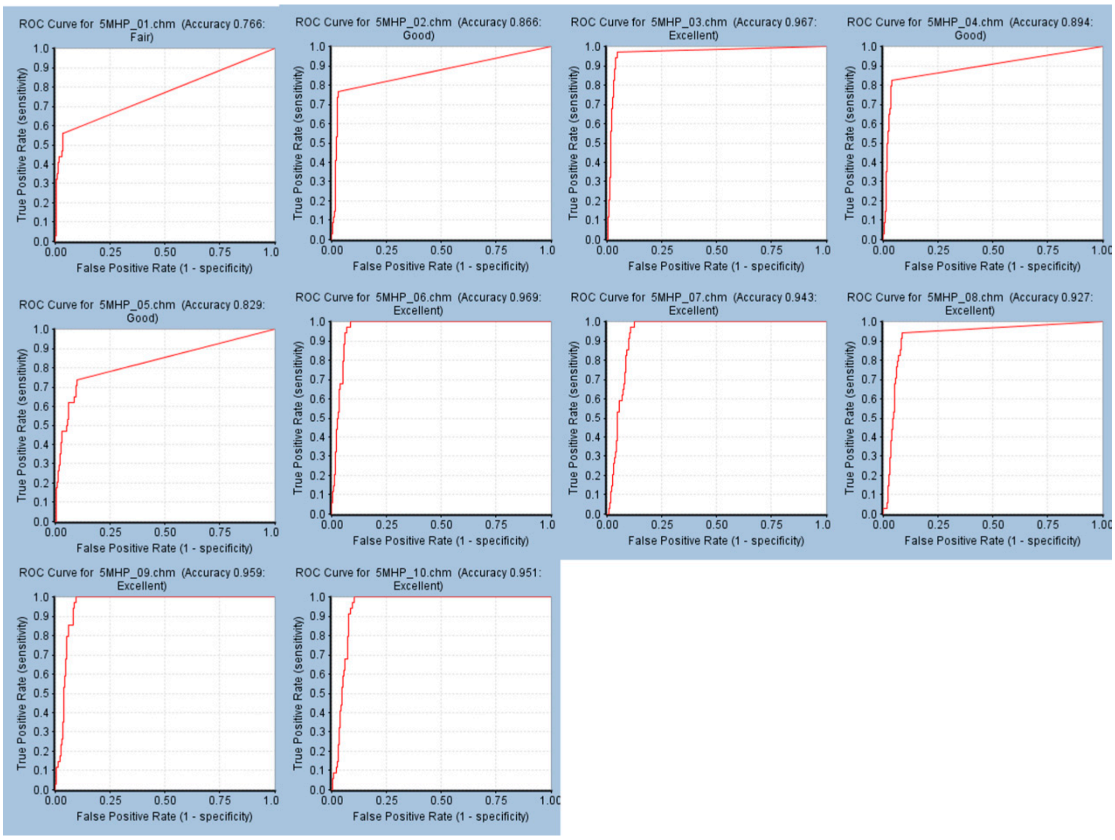

Figure S2. ROC curves of pharmacophore models 5MHP 01-10.
